# Indole-3-propionic acid suppresses prostate cancer by inducing cell cycle arrest and apoptosis associated with p53 activation

**DOI:** 10.3389/fonc.2026.1759301

**Published:** 2026-03-25

**Authors:** Yongneng Huang, Xinwei Liu, Yifan Wu, Shilin Li, Yang Shen, Hongyuan Wan, Yuwei Zhang, Ninghan Feng

**Affiliations:** 1 Department of Urology, Jiangnan University Medical Center, Wuxi, China; 2 Institute of Urology, Wuxi School of Medicine, Jiangnan University, Wuxi, China; 3 Department of Urology, Wuxi No. 2 People’s Hospital, Nanjing Medical University, Nanjing, China

**Keywords:** cell apoptosis, cell cycle, indole-3-propionic acid, p53-p21-RB, prostate cancer

## Abstract

**Introduction:**

Prostate cancer (PCa) poses a significant health burden worldwide, with castration-resistant progression representing a major therapeutic challenge. While gut microbiota metabolites have been increasingly linked to tumor development, the specific role of indole-3-propionic acid (IPA) in PCa remains unclear. This study explores the direct antitumor effects and molecular mechanisms of IPA in PCa progression.

**Methods:**

Using untargeted metabolomics, circulating metabolite profiles were characterized in serum samples from PCa patients and benign prostatic hyperplasia controls. The antitumor effects of IPA on PCa cells were evaluated with *in vitro* assays, including colony formation, wound healing, transwell migration, and flow cytometry for cell cycle and apoptosis analysis. *In vivo* efficacy of IPA was tested using a xenograft mouse model. Transcriptome sequencing (RNA-seq) and gene set enrichment analysis identified key biological processes. Western blot and quantitative PCR validated activation of the p53 signaling pathway.

**Results:**

Metabolomic analysis revealed markedly lower IPA levels in PCa patients, particularly in high-grade PCa. *In vitro* assays showed that IPA treatment effectively reduced PCa cell proliferation, migration, and invasion. *In vivo* studies with xenograft models demonstrated that IPA significantly slowed tumor growth. RNA-seq and gene set enrichment analysis pointed to cell cycle regulation as the main biological process affected by IPA. Flow cytometry confirmed that IPA caused cell cycle arrest and increased apoptosis in PCa cells. Mechanistic studies indicated that IPA specifically activates the p53-p21-RB signaling axis. Western blot and qPCR confirmed the increased expression of p53 and p21 after IPA treatment.

**Conclusion:**

IPA inhibits PCa cell proliferation, migration, and invasion, while inducing cell cycle arrest and apoptosis concurrent with the activation of the p53 signaling pathway, suggesting its potential as a new therapeutic option for PCa. These findings position IPA not only as a potential prognostic biomarker but also as a promising candidate for microbiota-based metabolic intervention in PCa management.

## 1 Introduction

Prostate cancer (PCa) represents a major global health threat to men, being the second most common cancer and accounting for approximately 1.5 million new cases and nearly 400,000 deaths annually (1–3). The pathogenesis of PCa involves multiple factors, including advanced age, genetic mutations, ethnicity, diet, and inflammation (4). Although second-generation androgen receptor axis inhibitors represent a primary treatment option, most metastatic cases progress to castration-resistant PCa following an initial therapeutic response (5). At this stage, effective treatment modalities are limited, resulting in an exceedingly poor prognosis. Consequently, novel therapeutic strategies are required to improve outcomes for patients with PCa.

Metabolic reprogramming is a well-established hallmark of cancer (6). This process involves alterations in extracellular metabolites that significantly influence malignant progression (7). In recent years, numerous microbiota-derived small-molecule metabolites have been identified as critical players in the initiation and progression of PCa, mediating tumor progression through mechanisms including epigenetic regulation, signaling pathways, and immune responses (8). For instance, gut microbiota-derived short-chain fatty acids (SCFAs) promote PCa progression by inducing cancer cell autophagy, promoting M2 macrophage polarization, or modulating IGF1 expression (9, 10). Meanwhile, acetate utilization has been reported to drive hormone therapy resistance in PCa through neuroendocrine differentiation (11). Notably, the roles of gut metabolites in PCa are complex and context-dependent. SCFAs exemplify this duality: while they can enhance the efficacy of anti-PD-1 immunotherapy by modulating T-cell receptor signaling in cytotoxic CD8^+^ T cells (12), they also exhibit protumoral properties, such as potentially suppressing antitumor immunity by inducing regulatory T cell differentiation or directly fueling tumor growth and therapy resistance (9, 13, 14). Furthermore, various tryptophan-derived indole metabolites exhibit anti-tumor activities through distinct mechanisms (15). Given that the circulatory system represents one of the largest reservoirs of metabolites in the body (16), systematic profiling of serum metabolites in cancer patients holds promise for identifying novel therapeutic targets.

Indole-3-propionic acid (IPA) is a crucial microbial metabolite derived from tryptophan metabolism in the gut (17), demonstrating extensive and vital pleiotropic effects in maintaining systemic homeostasis. Research has revealed that IPA exerts protective roles across multiple systems, including the nervous, cardiovascular, metabolic, and immune systems, primarily through its notable anti-inflammatory, antioxidant, and immunomodulatory properties (18). In the nervous system, IPA mediates neuroprotection via the microbiota-gut-brain axis, improving synaptic function, promoting neuroregeneration, and counteracting neuroinflammation and cognitive impairment, thereby showing potential in pathological contexts such as Alzheimer’s disease, autism susceptibility, and postoperative delirium (19–23). In the cardiovascular realm, IPA contributes to a protective mechanism termed the microbiota-IPA-heart axis by preserving cardiac function, inhibiting myocardial fibrosis, and mitigating atherosclerosis (24–26). Simultaneously, IPA serves as a key factor in maintaining intestinal barrier integrity; it suppresses gut dysbiosis, reinforces the epithelial and mucus barriers, and consequently reduces systemic endotoxemia and associated liver injury (27, 28). Its mechanisms of action broadly involve the activation of the aryl hydrocarbon receptor (AhR) and the inhibition of signaling pathways such as NF-κB and the NLRP3 inflammasome (29). Notably, in the field of tumor immunology, IPA epigenetically enhances T cell stemness and thereby improves immune checkpoint blockade (ICB) responsiveness across multiple cancer types, including melanoma, breast cancer, and colorectal cancer (30).

Given the highly complex and context-dependent roles of gut microbial metabolites, although IPA has demonstrated defined bioactivities in other cancer types, its specific function and underlying mechanisms in PCa remain to be elucidated. Therefore, systematically clarifying whether and how IPA influences PCa progression is essential to expand our understanding of the gut-prostate axis.

**Figure 1:**
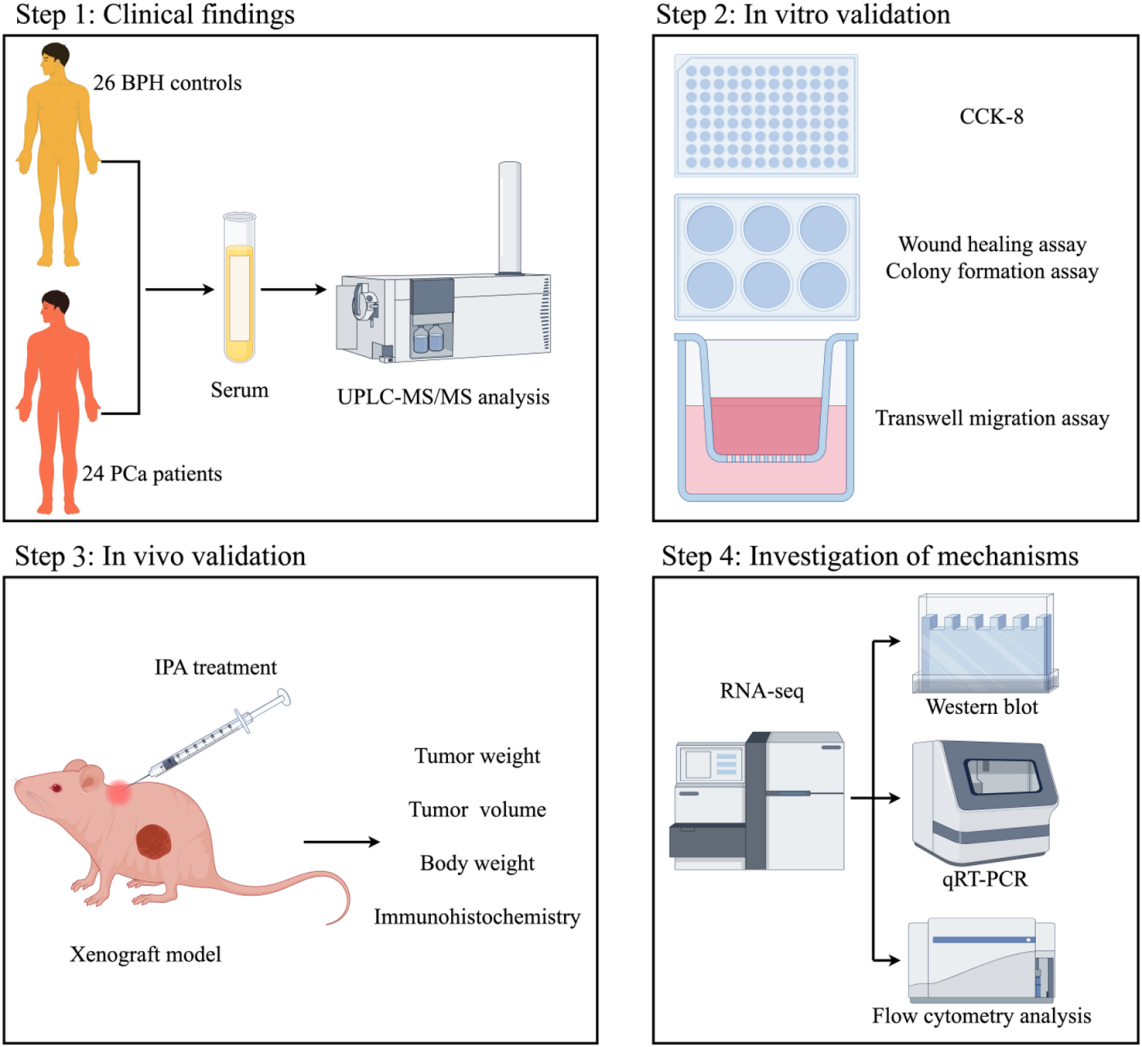
Schematic workflow of the study. The integrative research strategy comprises four key steps: (1) Collecting serum samples from 24 PCa patients and 26 BPH controls for untargeted metabolomic profiling; (2) Performing *in vitro* functional assays to validate the tumor-suppressive capacity of IPA using PCa cell lines; (3) Evaluating the anti-tumor efficacy of IPA *in vivo* employing a nude mouse xenograft model; (4) Investigating the underlying mechanisms through transcriptomic sequencing (RNA-seq) and molecular biological experiments, with functional validation by flow cytometry.

In this study, we will investigate the role and mechanisms of the microbiome-derived metabolite IPA in PCa, which may provide new therapeutic avenues for managing this disease. The overall experimental design and workflow of this study are illustrated in **Figure 1**.

## 2 Materials and methods

### 2.1 Sample collection

Serum samples were collected from patients with PCa and control subjects with benign prostatic hyperplasia (BPH) at the Jiangnan University Medical Center to investigate alterations in circulating metabolites. All participants provided written informed consent, and the human sample study received approval from the Ethics Committee of Jiangnan University Medical Center (No.2024-Y-44). Inclusion criteria for the PCa group were as follows (1): males aged 40–90 years (2); pathologically confirmed primary prostate adenocarcinoma (3); newly diagnosed and treatment-naïve. The BPH group consisted of age-matched males with pathologically confirmed BPH. Common exclusion criteria for both groups included (1): history of other malignancies (2); severe cardiovascular or cerebrovascular diseases (3); diagnosed endocrine disorders such as diabetes or thyroid dysfunction (4); severe dysfunction of vital organs (5); ethnic minorities with significantly different dietary habits (6); inability to cooperate.

### 2.2 Sample collection and processing

Following a minimum 8-hour fasting period, peripheral venous blood was collected from all participants using vacuum serum collection tubes containing clot activator. Tubes were maintained at room temperature for 30–60 minutes to allow complete clot formation, then transferred to 4°C and processed within 2 hours. The processing protocol consisted of (1): centrifugation at 4°C and 3000 × g for 15 minutes (2); careful aspiration of the serum supernatant (3); aliquoting into pre-labeled sterile 1.5 mL cryovials (4); immediate storage at −80°C until subsequent metabolomic analysis. All samples were protected from repeated freeze-thaw cycles to preserve metabolite integrity.

### 2.3 Sample preparation and UPLC–MS/MS analysis

Serum samples were extracted with 600 μL of methanol (−20°C) and 100 mg of glass beads, followed by vortexing for 60 s. The mixture was centrifuged at 12,000 rpm and 4°C for 10 min. A total of 400 μL of the supernatant was collected and concentrated to dryness using a vacuum concentrator. The residue was reconstituted in 100 μL of 30% methanol, then filtered through a 0.22 μm membrane, and the filtrate was transferred to an LC–MS vial. The LC–MS analysis was carried out using an EXionLC™ Liquid Chromatography system (AB SCIEX, United States) coupled with an AB6500 Plus mass spectrometer (AB SCIEX, United States). Separation was achieved on an ACQUITY UPLC^®^ BEH C18 column (2.1 × 100 mm, 1.7 μm; Waters, United States) maintained at 40°C. The injection volume was 5 μL. The mobile phase consisted of (A) 0.01% formic acid in water and (B) acetonitrile. The gradient elution program was as follows: 0–4 min, 25% B; 4–9 min, 25–30% B; 9–14 min, 30–36% B; 14–18 min, 36–38% B; 18–24 min, 38–50% B; 24–32 min, 50–75% B; 32–33 min, 75–90% B; 33–35.5 min, 90–25% B. The flow rate was set at 0.25 mL/min. Mass spectrometric detection was performed using an electrospray ionization (ESI) source in negative ion mode. The ion source temperature was 500°C, the ion spray voltage was −4,500 V, the collision gas pressure was 6 psi, the curtain gas pressure was 30 psi, and both the nebulizing gas and auxiliary gas were set at 50 psi. Multiple reaction monitoring (MRM) was used for data acquisition.

### 2.4 Cell culture

The human PCa cell lines PC3 (Cell Bank of the Chinese Academy of Sciences) and 22RV1 (Wuhan Procell Life Science & Technology Co., Ltd.) were used in this study. Cells were maintained in RPMI-1640 medium (Gibco, 11875500BT) supplemented with 10% heat-inactivated fetal bovine serum (Celligent, CG0430B) and 1% antibiotic-antimycotic solution (Gibco, 15410-122) at 37°C in a humidified incubator with 5% CO_2_. When cell density reached 80%, PC3 and 22RV1 cells were seeded onto different types of plates and subjected to various treatment conditions for subsequent experiments. IPA (MCE, HY-113099) was dissolved in DMSO. The experimental protocol ensured that the concentration of DMSO in experimental groups was equivalent to that in the control group.

### 2.5 Mice

Four-week-old male BALB/c nude mice (GemPharmatech Co., Ltd.; Stock No. D000521) were housed in the barrier facility of the Experimental Animal Center at Jiangnan University. Mice were maintained in individually ventilated cages under controlled conditions (temperature, 22 ± 2°C; humidity, 50 ± 10%; 12-hour light/dark cycle) with ad libitum access to autoclaved chow and sterile water. All animal handling procedures adhered to the guidelines established by the National Institutes of Health and were approved by the Animal Welfare and Ethics Committee of Jiangnan University (JN.NO20251114B024177[189]), Wuxi, China.

### 2.6 *In vivo* animal studies

All mice were uniquely identified with ear tags and randomly assigned to experimental groups using a random number table method. A single-blind study design was implemented in which the experimental operators were blinded to group assignments, while dedicated personnel were responsible for drug preparation. Treatments were administered strictly according to the assigned numerical identifiers. In the PCa xenograft model, 4-week-old male BALB/c nude mice were anesthetized with 2% isoflurane in oxygen and subcutaneously injected in the left flank with a suspension containing 5×10^6^ 22RV1 cells in a 1:1 mixture of 1640 medium and Matrigel, with a total volume of 200 μL. When tumor volume reached approximately 80 mm³, the mice were randomly divided into three groups: control group (subcutaneous injection of 10% DMSO in PBS), low-dose IPA group (15 mg/kg/day, s.c.), and high-dose IPA group (60 mg/kg/day, s.c.). All drug injections were administered subcutaneously (s.c.) daily in the dorsal cervical region. Tumor length and width were measured every 4 days using digital calipers, and tumor volume was calculated using the formula: Volume = (Length × Width²)/2. The experiment continued for 17 days. At the experimental endpoint, mice were euthanized by CO_2_ inhalation at a flow rate of 30% of the chamber volume per minute for 5 minutes. Tumors were completely excised and weighed for subsequent histological and molecular biological analyses.

### 2.7 RNA-seq experiments

Total RNA extracted using TRIzol from all samples with confirmed integrity (RIN >7.0) was subjected to poly(A) selection using Oligo(dT) beads, followed by magnesium-based RNA fragmentation, double-stranded cDNA synthesis, and construction of 300 bp insert libraries, which were sequenced on the Illumina NovaSeq 6000 platform (LC-Bio Technology Co., Ltd., Hangzhou) with paired-end 150 bp reads; raw data were quality-controlled and adapter-trimmed using fastp, then aligned to the GRCh38 human genome with HISAT2, and transcript abundance was quantified by StringTie in FPKM units, with differentially expressed genes identified using edgeR (fold change >2 or <0.5, *p* < 0.05).

### 2.8 Quantitative real-time PCR

RNA extraction from 22RV1 cells (each sample used 3 × 10^6 cells) was carried out using a FastPure^®^ Cell/Tissue Total RNA Isolation Kit V2 (Vazyme, RC112−01) according to the manufacturer’s protocol. RNA was reverse transcribed into cDNA using a HiScript IV All-in-One Ultra RT SuperMix for qPCR (Vazyme, R433-01). qPCR was carried out in an Applied Biosystems 7500 PCR system (Thermo Fisher, USA) using a ChamQ Universal SYBR^®^ qPCR Master Mix (Vazyme, Q711−02). The RT-qPCR protocol consisted of the following steps: Initiation at 95˚C for 30 seconds, followed by 40 cycles at 95˚C for 10 seconds and 60˚C for 30 seconds. Relative quantification of gene expression was carried out using the 2-ΔΔCq method, with GAPDH as the endogenous control. All qRT-PCR primers were synthesized by Sangon Biotech (Shanghai). A list of the qRT-PCR primers can be found in **Table 1**. The results were analyzed using a previously reported method (31).

**Table 1:** Primer sequences used for qPCR analysis.

Primer	Sequence (5′→3′)
CDKN1A Forward	GCGACTGTGATGCGCTAATG
CDKN1A Reverse	GAAGGTAGAGCTTGGGCAGG
GAPDH Forward	GGAGCGAGATCCCTCCAAAAT
GAPDH Reverse	GGCTGTTGTCATACTTCTCATGG
TP53 Forward	CCTCTCCCCAGCCAAAGAAG
TP53 Reverse	CTTCAGGTGGCTGGAGTGAG
CCNA2 Forward	GGATGGTAGTTTTGAGTCACCAC
CCNA2 Reverse	CACGAGGATAGCTCTCATACTGT
CCNB2 Forward	TGCTCTGCAAAATCGAGGACA
CCNB2 Reverse	GCCAATCCACTAGGATGGCA
CCNB1 Forward	TCGCATCAAACTCTCTGGCTA
CCNB1 Reverse	TGAGCGACTAAACTCACCACT

### 2.9 Western blot analysis

Cells were lysed with lysis buffer at a ratio of 200 μL per 1×10^6^ cells. The lysates were centrifuged at 14,000 × g for 20 minutes at 4°C to collect the supernatant. Protein concentration was determined using a BCA protein assay kit (Beyotime, P0010). Equal amounts of protein (20 μg per lane) were separated by 10% SDS-PAGE and transferred onto polyvinylidene fluoride (PVDF) membranes. The membranes were blocked with 5% bovine serum albumin (Biofoxx, 9048-46-8) in Tris-buffered saline containing 0.1% Tween-20 (BioSharp, 9005-64-5) for 1 hour at room temperature, followed by incubation with specific primary antibodies overnight at 4°C. After washing, the membranes were incubated with the corresponding secondary antibodies for 1 hour at room temperature. Protein bands were visualized using a chemiluminescence imaging system. The antibodies used are: anti-p53 (1:2,000; Cat No. 10442-1-AP; Wuhan Servicebio Technology), anti-phospho-p53 (Ser15) (1:2,500; Cat No. 80195-1-RR; Wuhan Servicebio Technology), anti-p21 (1:2,000; Cat No. 10355-1-AP; Wuhan Servicebio Technology), anti-phospho-RB (Ser780) (1:4,000; Cat No. 84692-1-RR, Wuhan Servicebio Technology), anti-β-actin (1:2,000; Cat No. GB12001; Wuhan Servicebio Technology), and anti-GAPDH (1:2,000; Cat No. 10494-1-AP; Wuhan Servicebio Technology). Anti-rabbit IgG (1:5,000; cat. no. GB23303, Wuhan Servicebio Technology), anti-mouse IgG (1:5,000; cat. no. GB23301, Wuhan Servicebio Technology).

### 2.10 Colony formation assay

To assess colony-forming ability, 500 cells were seeded into each well of 6-well plates and cultured in complete medium at 37°C with 5% CO_2_ for two weeks. Following incubation, cells were fixed with methanol for 30 minutes and stained with 0.2% crystal violet solution (Solarbio, G1062). Colonies containing more than 50 cells were counted for quantitative analysis.

### 2.11 Wound healing assay

The wound healing assay was performed to evaluate the migration ability of PCa cells. 22RV1 and PC3 cells were seeded in 6-well plates at a density of 5 × 10^5 cells per well and cultured in complete medium containing 10% fetal bovine serum at 37°C with 5% CO_2_ until reaching full confluence. A sterile 200 μL pipette tip was used to create a straight wound in the cell monolayer. The detached cells were removed by gently washing three times with PBS. The medium was then replaced with low-serum medium containing 1% fetal bovine serum to minimize the effect of cell proliferation. Wound closure was observed and recorded at 0, 24, and 48 hours using an inverted microscope. The changes in wound area were analyzed using ImageJ software, and the migration rate was calculated as: (Initial wound area - Wound area at specific time point)/Initial wound area × 100%. All experiments were independently repeated three times.

### 2.12 Transwell migration assay

Cell migration was assessed using Transwell^®^ cell culture inserts (Corning, 3422). 22RV1 and PC3 cells (5×10^4^ cells/well) were suspended in serum-free 1640 medium and seeded into the upper chambers (200 μL). The lower chambers were filled with 1640 medium containing 20% fetal bovine serum as a chemoattractant (600 μL). After 24 hours of incubation at 37°C with 5% CO_2_, non-migrated cells on the upper surface of the membrane were carefully removed using cotton swabs. Cells that had migrated to the lower surface were fixed with 4% paraformaldehyde for 20 minutes and stained with 0.1% crystal violet (Solarbio, G1062) for 30 minutes. Five random fields per membrane were imaged under an inverted microscope and counted. The experiment was independently repeated three times.

### 2.13 Proliferation assay

Cell proliferation was assessed using the CCK-8 assay (Beyotime Biotechnology). 22RV1 and PC-3 cells under various treatment conditions were seeded in 96-well plates. At the indicated time points, 10% CCK-8 solution was added to each well and incubated for 2 hours. Absorbance was measured at 450 nm.

### 2.14 Flow cytometry analysis

Apoptosis was evaluated by flow cytometry using an Annexin V-FITC/PI double-staining assay according to the manufacturer’s protocol (Beyotime, C1062L). Cell cycle distribution was determined through propidium iodide DNA staining following the kit instructions (Beyotime, C1052). Flow cytometric analyses were conducted on a CytoFlex system (Beckman Coulter). Apoptosis data were processed using FlowJo software (v10.8.1, Treestar, Inc., San Carlos), and cell cycle profiles were analyzed with ModFit LT (v5.0, Verity Software House, Topsham).

### 2.15 Immunohistochemical analysis

Subcutaneous xenograft tumors were dissected after euthanasia and fixed in 4% paraformaldehyde for 24 hours. The tissues were dehydrated through a graded ethanol series, cleared in xylene, and embedded in paraffin blocks. Sequential sections of 4 μm thickness were cut using a rotary microtome. For hematoxylin and eosin (H&E) staining, sections were deparaffinized in xylene, rehydrated through a graded ethanol series, stained with hematoxylin for 5 minutes, differentiated in acid ethanol, counterstained with eosin for 2 minutes, and finally mounted with neutral balsam. For immunohistochemical (IHC) analysis, antigen retrieval was performed using citrate buffer (pH 6.0) under high-pressure heating. Endogenous peroxidase activity was blocked with 3% hydrogen peroxide, and nonspecific binding sites were blocked with 5% bovine serum albumin. Sections were incubated with primary antibodies at 4°C overnight, followed by incubation with HRP-conjugated secondary antibodies at room temperature for 30 minutes. Color development was performed using a DAB substrate kit, and nuclei were counterstained with hematoxylin before mounting with neutral balsam. All slides were examined and imaged using a Nikon Eclipse Ci microscope. Quantitative analysis was performed using ImageJ software (Version 1.53, National Institutes of Health, USA). Negative controls were processed by replacing the primary antibodies with PBS.

### 2.16 Statistical analysis

Unless otherwise stated, all quantitative data in this study are presented as mean ± standard deviation (SD), with specific sample sizes (n values) detailed in the corresponding method descriptions and figure legends. For comparisons between two groups that meet the assumptions of normal distribution and homogeneity of variance, an unpaired two-tailed Student’s t-test was employed. For comparisons involving three or more groups, one-way analysis of variance (ANOVA) is used. All statistical analyses were performed using GraphPad Prism version 9.0 (GraphPad Software, San Diego, CA, USA). Significance levels are denoted as follows: *p < 0.05, **p < 0.01, ***p < 0.001, and ns indicating not statistically significant.

## 3 Results

### 3.1 Altered circulating metabolites in PCa patients with significantly reduced levels of IPA

**Figure 2:**
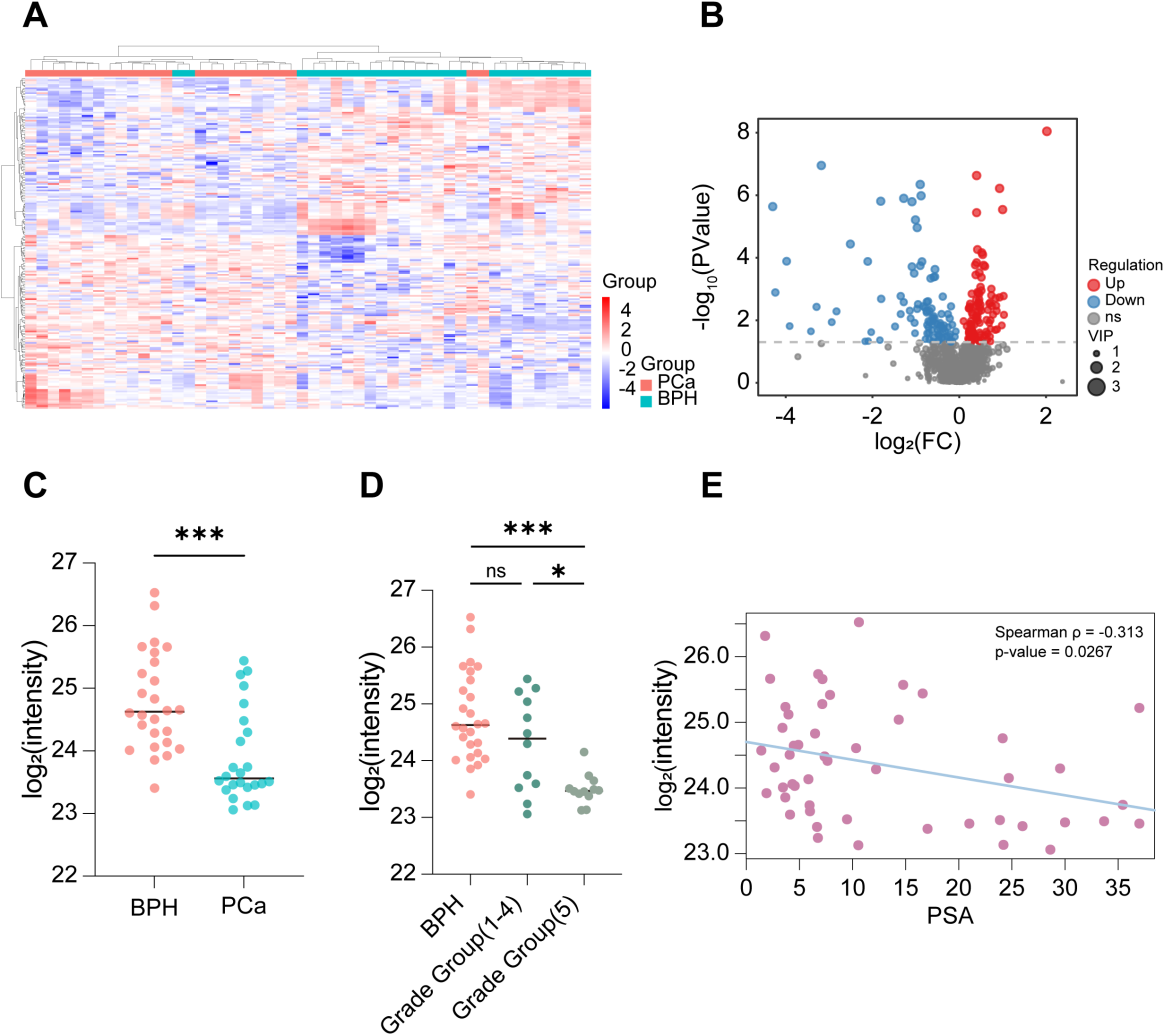
IPA decreases in the serum of PCa patients. **(A)** Hierarchical clustering heatmap of differentially abundant metabolites, showing distinct clustering of BPH and PCa samples. **(B)** Volcano plot of differentially abundant metabolites, identifying 107 upregulated and 97 downregulated metabolites in PCa compared to BPH (criteria: p-value < 0.05). **(C)** The relative intensity of Indole-3-propionic acid (IPA) in the serum of the PCa (n = 24) and BPH (n = 26) groups. **(D)** According to the Grade Group system, PCa patients were categorized into Grade Groups 1-5, and the relative intensity of serum IPA was significantly lower in Grade Group 5 compared to Grade Groups 1-4. **(E)** Spearman’s rank correlation analysis revealed a significant negative correlation between PSA and log2(IPA intensity) (ρ = -0.313, *p* = 0.027), (n = 50). * denotes p < 0.05, *** denotes p < 0.001, and ns indicates not significant (p ≥ 0.05).

To investigate alterations in circulating metabolites among PCa patients, we performed untargeted metabolomics analysis on serum samples from 26 PCa patients and 24 patients with benign prostatic hyperplasia (BPH). Orthogonal Partial Least Squares-Discriminant Analysis (OPLS-DA) revealed a clear distinction between the PCa and BPH groups, indicating unique circulating metabolite profiles (**Supplementary Figures S1A, B**). The heatmap of significantly altered secondary metabolites illustrated a pronounced separation between PCa patients and BPH controls (**Figure 2A**). Further comparative analysis identified 204 metabolites that were significantly altered in the PCa group relative to the BPH group, of which 107 were upregulated and 97 were downregulated (**Figure 2B**).

We focused on gut microbiota-derived metabolites and found a significant reduction in serum IPA levels in PCa patients (**Figure 2C**). Based on the Gleason score-based PCa grading system (Grade groups 1-5) (32), we found that patients with Grade group 5 tumors showed significantly lower IPA levels compared to those with Grade groups 1-4 (**Figure 2D**). Spearman’s rank correlation analysis revealed a significant negative correlation between PSA and log_2_ (IPA intensity) (ρ = -0.313, *p* = 0.027, n = 50) (**Figure 2E**).

In summary, our clinical data demonstrate that circulating IPA levels are significantly lower in PCa patients than in BPH controls, with this reduction being more pronounced in more aggressive tumors. These findings suggest that IPA may serve as a protective metabolite, and its decrease could be associated with PCa progression.

### 3.2 IPA treatment inhibits the viability of PCa cells

**Figure 3:**
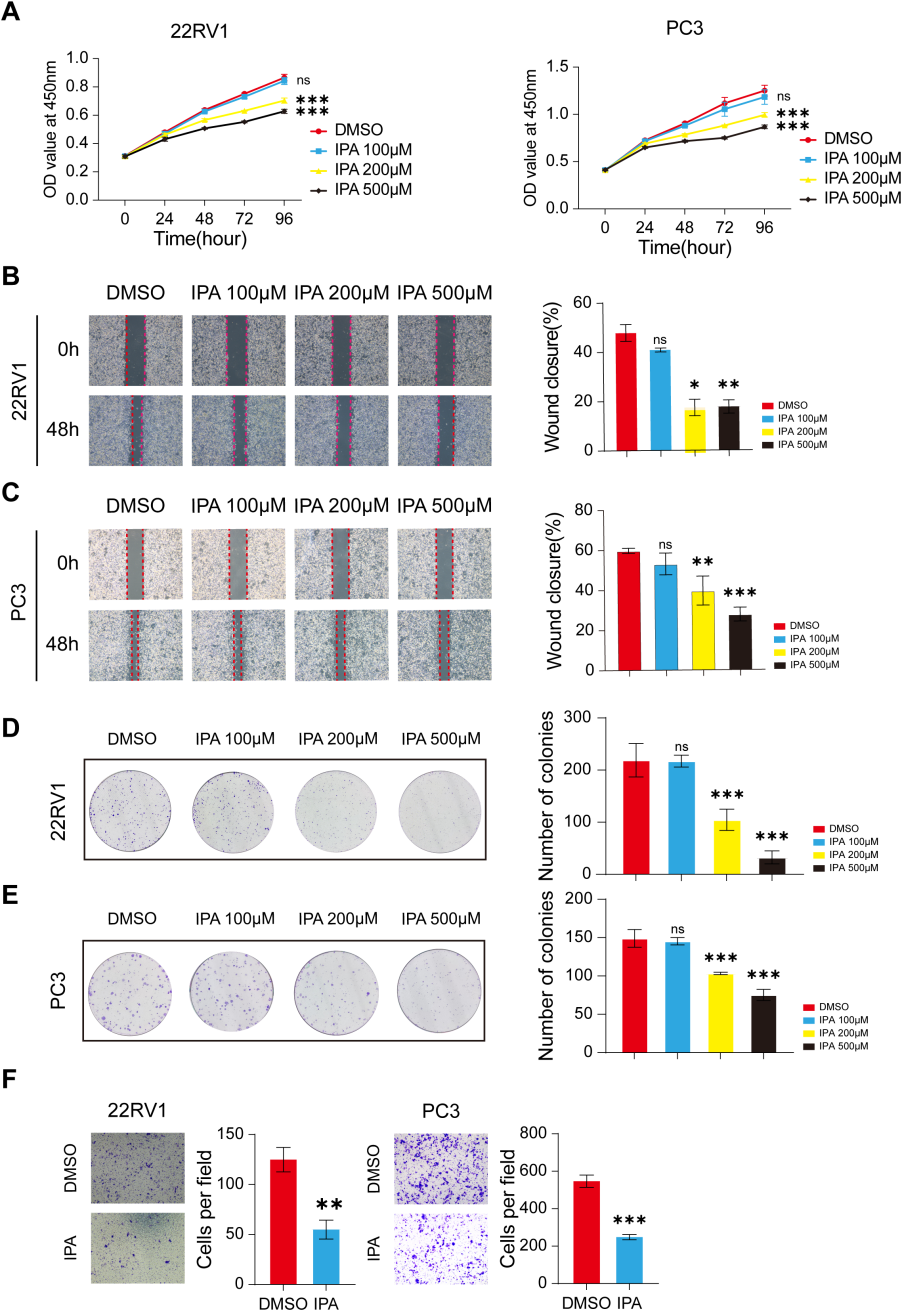
IPA treatment inhibits the viability of PCa cells. **(A)** The proliferation of 22RV1 and PC-3 cells treated with DMSO or IPA was evaluated at the indicated concentrations and time points using the CCK-8 assay (n = 5). **(B, C)** The migratory ability of 22RV1 **(B)** and PC3 **(C)** cells after treatment with DMSO or IPA at the indicated concentrations was evaluated using the wound healing assay (n = 3). **(D, E)** The colony formation assay was employed to assess the clonogenic capacity of 22RV1 **(D)** and PC3 **(E)** cells following exposure to DMSO or IPA at the indicated concentrations (n=3). **(F)** The invasive ability of 22RV1 and PC3 cells was analyzed using the transwell invasion assay with Matrigel after treatment with DMSO or 200μM IPA (n=3). * denotes p < 0.05, ** denotes p < 0.01, *** denotes p < 0.001, and ns indicates not significant (p ≥ 0.05).

To investigate the potential direct effects of IPA on tumor cells, we conducted *in vitro* functional analyses using two PCa cell lines, 22RV1 and PC3. First, we used the CCK-8 assay to examine the effect of IPA on cell proliferation. The results showed that IPA treatment significantly inhibited the viability of 22RV1 and PC3 cells in a concentration-dependent manner (**Figure 3A**). The cells were treated with various concentrations of IPA for specified durations, and the functional assays demonstrated that IPA significantly inhibited malignant phenotypes in PCa cells. A wound healing assay revealed that IPA effectively suppressed the migration of both 22RV1 and PC3 cells in a concentration-dependent manner (**Figures 3B, C**). Colony formation assays indicated that IPA treatment significantly reduced the clonogenic ability of PCa cells in a dose-dependent manner (**Figures 3D, E**). Based on these dose-response analyses, 200 μM was identified as the minimal concentration that produced significant inhibition in both assays and was therefore selected for all subsequent *in vitro* experiments. Regarding metastatic potential, Transwell migration assays demonstrated that IPA treatment significantly decreased the number of migrating 22RV1 and PC3 cells, suggesting its potent inhibitory effect on the migratory capacity of PCa cells (**Figure 3F**).

Collectively, these results indicate that IPA, at certain concentrations, directly inhibits proliferation, clonogenesis, and migration of PCa cells *in vitro*, underscoring its potential therapeutic value in suppressing PCa progression.

### 3.3 IPA treatment suppresses PCa progression *in vivo*

**Figure 4:**
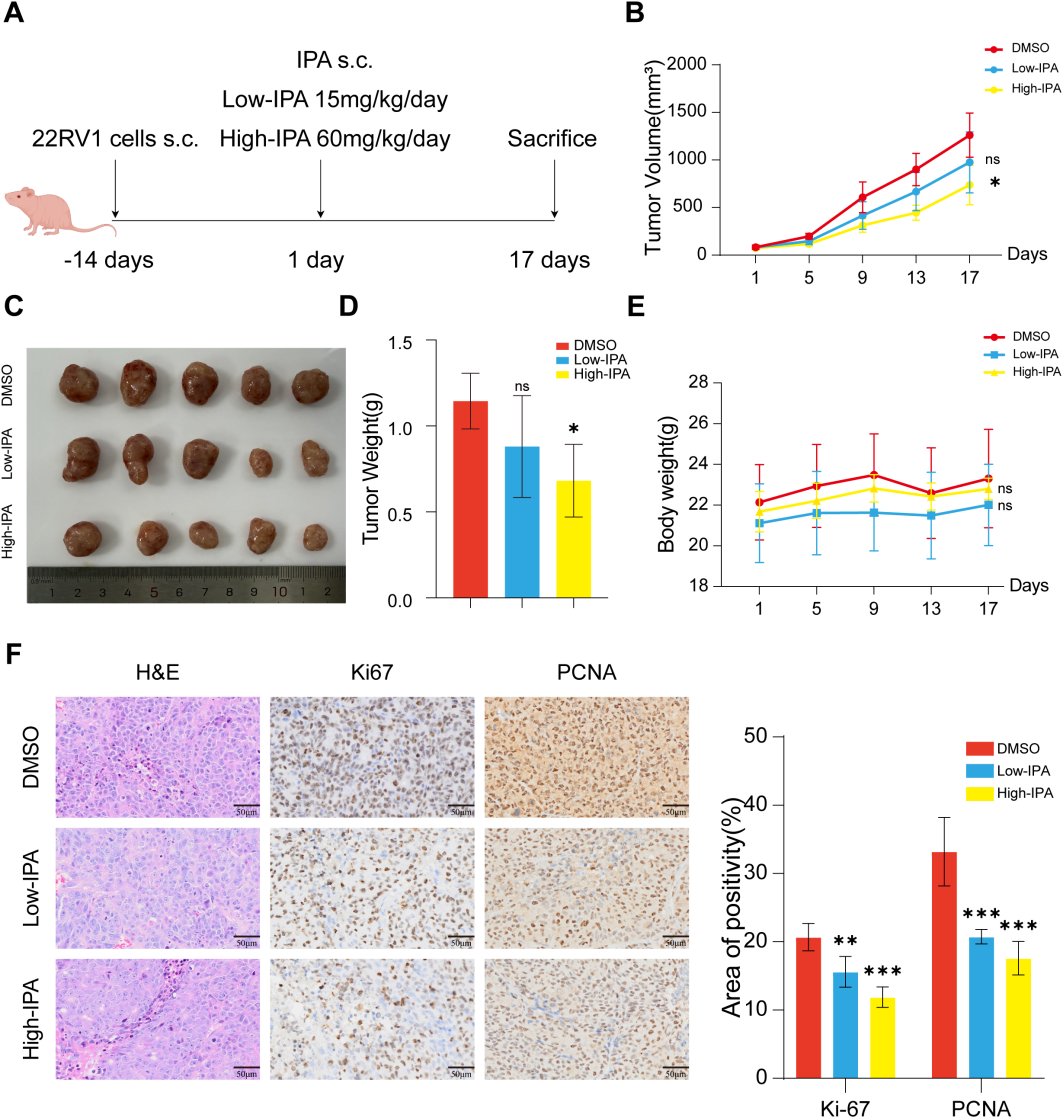
IPA treatment suppresses PCa progression *in vivo*. **(A)** Experimental design for the 22RV1 xenograft model in Babl/c nude mice and IPA administration schedule (n = 5). s.c.: subcutaneous injection. **(B–D)** The alterations in tumor volume were assessed over the 17-day IPA treatment period **(B)**, tumors were excised and photographic images were captured **(C)**, and tumor weight was determined **(D)**. **(E)** Body weight changes of mice in each group. **(F)** Histological analysis of prostate cancer xenografts was shown by H&E staining with additional immunohistochemical staining for Ki67 and PCNA. H&E: hematoxylin and eosin; PCNA: proliferating cell nuclear antigen. * denotes p < 0.05, ** denotes p < 0.01, *** denotes p < 0.001, and ns indicates not significant (p ≥ 0.05).

Based on the observed reduction in circulating IPA levels in PCa patients, combined with our *in vitro* findings that IPA treatment inhibits PCa cell viability, we hypothesized that IPA supplementation might delay PCa progression *in vivo*. To test this hypothesis, we established a BALB/c nude mouse xenograft model by subcutaneously inoculating PCa cells into mice and conducted an *in vivo* intervention study, with the drug administration scheme illustrated in **Figure 4A**. The animals were randomly assigned to three groups: Group A (control, DMSO), Group B (Low-IPA, 15 mg/kg/day), and Group C (High-IPA, 60 mg/kg/day). We observed that, compared to the control group, high-IPA supplementation significantly inhibited the growth rate of subcutaneous tumors and reduced tumor burden (**Figures 4B–D**). Although the Low-IPA group exhibited a reduction in average tumor weight, this difference did not achieve statistical significance. No significant differences in body weight were noted among the groups throughout the experimental period (**Figure 4E**). After 17 days of continuous IPA administration, all mice survived without exhibiting signs of decreased vitality or other toxic reactions. On day 17, the mice were euthanized, and 22RV1 tumor tissues were collected. Immunohistochemical analysis revealed that the IPA-treated group exhibited decreased expression levels of Ki67 and Proliferating Cell Nuclear Antigen (PCNA) (**Figure 4F**).

In summary, in the BALB/c nude mouse xenograft model, IPA treatment significantly suppressed tumor growth, suggesting potential antitumor mechanisms beyond CD8^+^ T cell involvement.

### 3.4 Transcriptomic profiling identifies cell cycle arrest as a key response to IPA treatment

**Figure 5:**
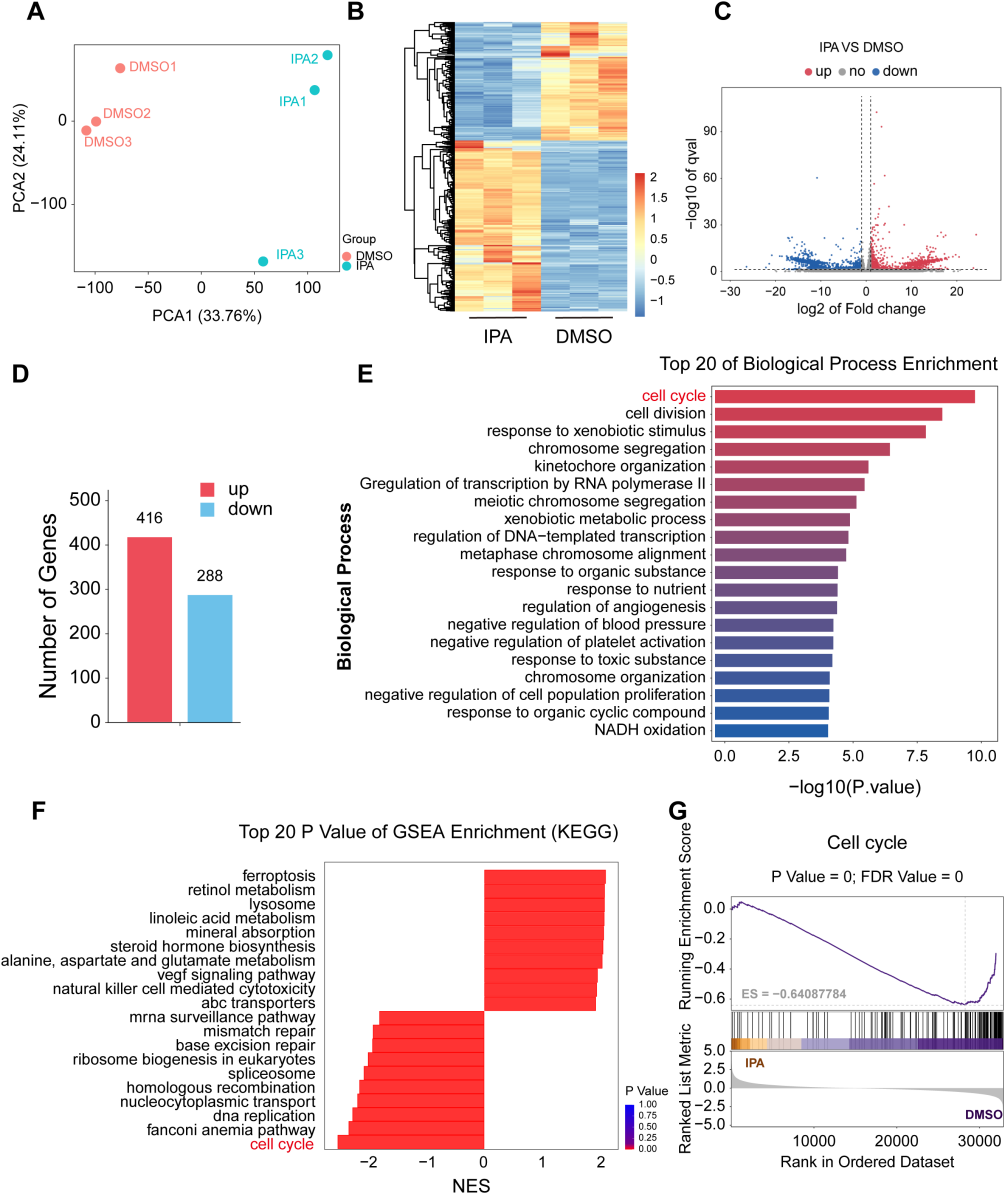
Transcriptomic profiling identifies cell cycle arrest as a key response to IPA treatment. **(A, B)** RNA-seq analysis of 22RV1 cells treated with DMSO or 200 μM IPA. **(A)** Principal component analysis (PCA) plot showing distinct global transcriptomic profiles. **(B)**Hierarchical clustering heatmap of differentially expressed genes. **(C, D)** Volcano plot and bar plot summarizing the number of upregulated (416) and downregulated (288) genes. **(E)** Gene Ontology (GO) analysis showing the top 20 significantly enriched biological processes. **(F)** The top 20 enriched gene sets from GSEA ranked by p-value. **(F)** GSEA analysis showed a decrease in cell cycle pathway enrichment after IPA treatment. GSEA: gene set enrichment analysis.

To elucidate the mechanism by which IPA inhibits PCa cell activity, we conducted RNA-seq analysis on the PCa cell line 22RV1 co-cultured with 200 μM IPA. The results indicated that IPA induced significant alterations in the global gene expression profile, as evidenced by the clear separation between treatment and control groups in both principal component analysis (PCA) and hierarchical clustering heatmaps (**Figures 5A, B**). Differential expression analysis revealed 704 significantly altered genes, comprising 416 upregulated and 288 downregulated genes (**Figures 5C, D**). Gene Ontology (GO) enrichment analysis of these differentially expressed genes identified “cell cycle” as the most significantly enriched term among biological processes (**Figure 4E**). In alignment with this finding, Gene Set Enrichment Analysis (GSEA) further confirmed a significant suppression of the “cell cycle” pathway (**Figures 5F, G**). Collectively, these data suggest that the core of IPA-triggered transcriptional reprogramming is centered on the regulation of cell cycle progression.

### 3.5 IPA activates the p53-p21-RB signaling axis concurrent with G0/G1 phase cell cycle arrest and apoptosis

**Figure 6:**
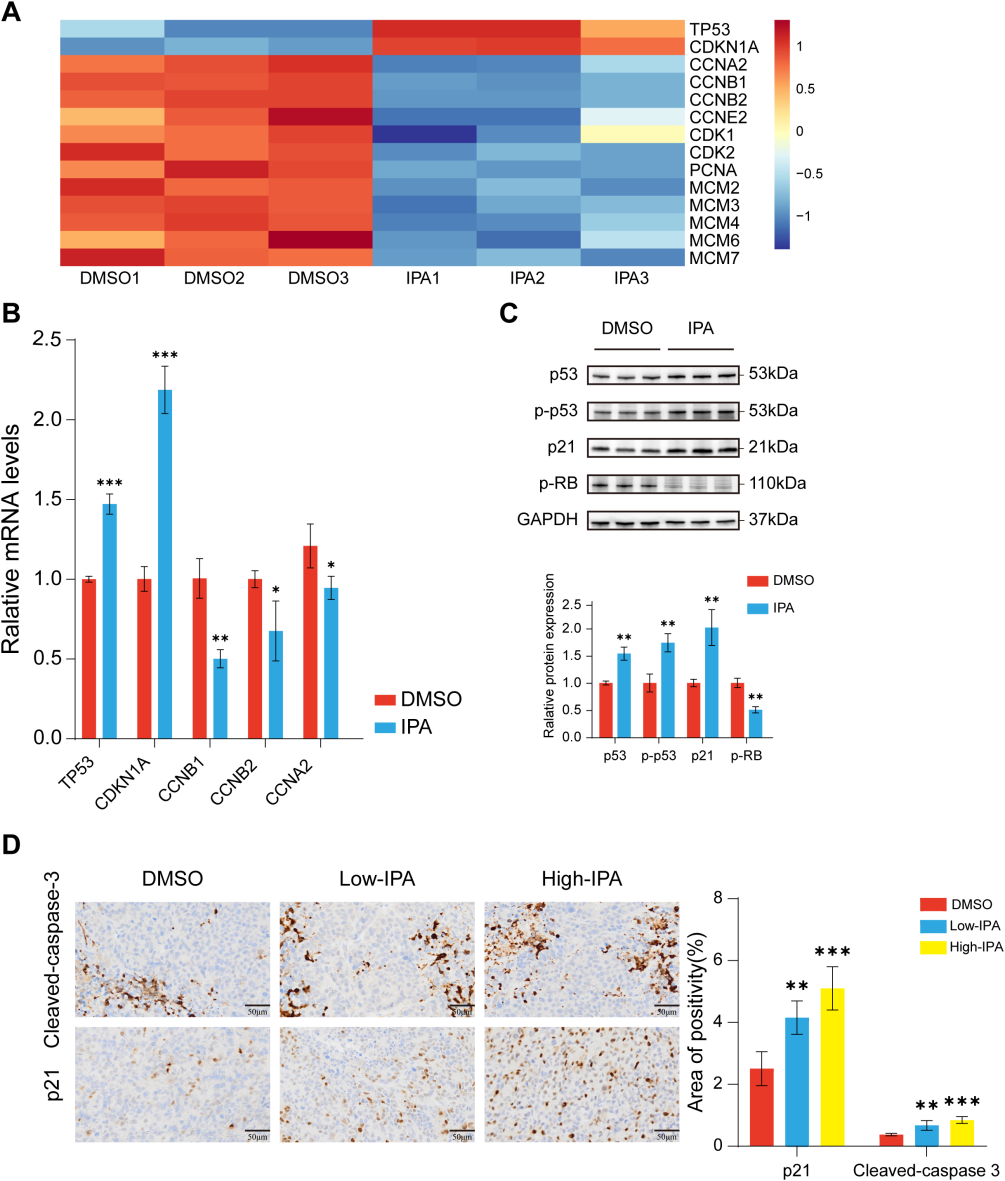
IPA activates the p53-p21-RB signaling axis **(A)** Heatmap of RNA-seq data showing the expression patterns of key cell cycle-related genes in 22RV1 cells treated with DMSO or 200μM IPA. **(B)** RT-qPCR validation of the mRNA expression levels of cell cycle regulators in 22RV1 cells after IPA treatment. **(C)** Western blot analysis (left) and quantitative results (right) demonstrating the protein expression levels of p53, phospho-p53, p21, and phospho-RB in 22RV1 cells following 200 μM IPA treatment. **(D)** Immunohistochemical analysis of xenograft tumor tissues for p21 and cleaved caspase-3. * denotes p < 0.05, ** denotes p < 0.01, *** denotes p < 0.001, and ns indicates not significant (p ≥ 0.05).

Transcriptomic profiling further elucidated the mechanism of IPA action, showing a coordinated upregulation of cell cycle arrest genes (including *TP53* and *CDKN1A*) and concurrent downregulation of genes driving cell cycle progression (e.g., *CCNB1*, *CCNB2*, *CCNA2*) (**Figure 6A**). This expression pattern was validated through RT-qPCR in 22RV1 cells (**Figure 6B**). Western blot analysis corroborated the impact of IPA treatment on cell cycle regulatory proteins. Specifically, IPA treatment significantly elevated the expression levels of p53, phosphorylated p53, and p21, while concurrently reducing p-RB expression in 22RV1 cells (**Figure 6C**). Furthermore, immunohistochemical analysis of xenograft tumor tissues indicated that IPA treatment upregulates the expression of p21 and cleaved caspase-3 (**Figure 6D**).

**Figure 7:**
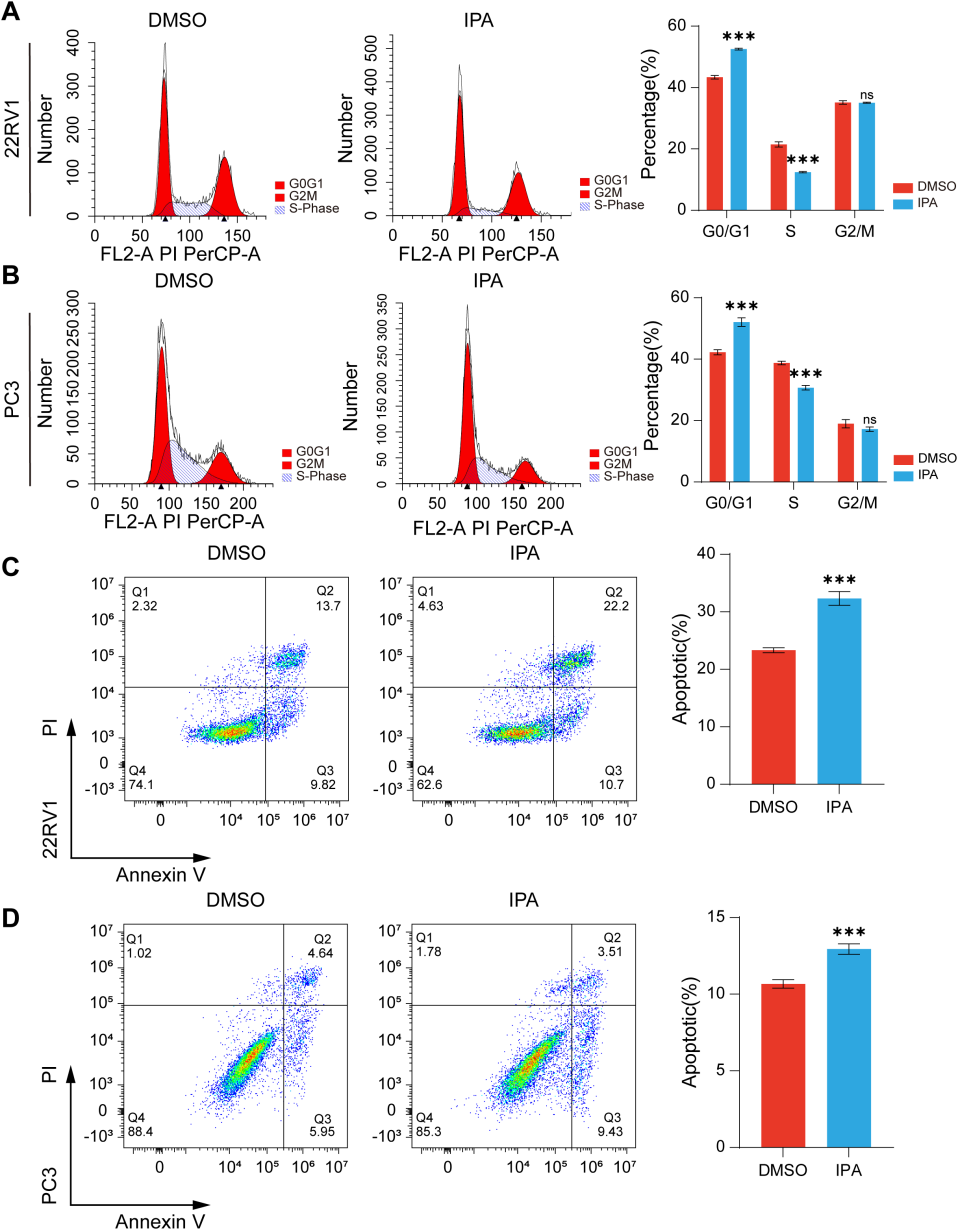
IPA treatment induces G0/G1 phase cell cycle arrest and apoptosis. **(A, B)** Cell cycle distribution analyzed by flow cytometry in 22RV1 **(A)** and PC3 **(B)** cells treated with DMSO or IPA (200 μM) for 48 hours, showing the percentage of cells in G0/G1, S, and G2/M phases. **(C, D)** Apoptosis rate assessed by flow cytometry in 22RV1 **(C)** and PC3 **(D)** cells treated with DMSO or IPA (200 μM) for 48 hours. *** denotes p < 0.001, and ns indicates not significant (p ≥ 0.05).

We further examined the effect of IPA on cell cycle progression in PCa cells. Flow cytometry analysis revealed that IPA treatment induced G0/G1 phase cell cycle arrest in both PC3 and 22RV1 cells (**Figures 7A, B**). Considering the essential role of TP53 in regulating apoptosis (33), we assessed apoptotic rates and found that IPA treatment significantly enhanced apoptosis in both 22RV1 and PC3 cells (**Figures 7C, D**).

Together, these data indicate that IPA treatment activates the TP53 signaling pathway, upregulates P21 expression, induces cell cycle arrest, and promotes apoptosis, thereby ultimately suppressing the viability of PCa cells. These findings underscore IPA’s potential to inhibit PCa progression by directly targeting pathways related to tumor cell cycle regulation and apoptosis.

## 4 Discussion

This investigation reveals a significant association between reduced circulating IPA levels and disease advancement in PCa patients. Our experimental data, obtained from both *in vitro* and animal studies, demonstrate that IPA exerts tumor-suppressive effects in a dose-responsive manner. Notably, this study indicates that IPA can directly target PCa cells and inhibit their activity independently of antitumor immune mechanisms. Collectively, our findings demonstrate that IPA treatment induces cell cycle arrest and apoptosis, processes associated with the activation of the p53-p21-RB axis, thereby contributing to tumor suppression.

In recent years, there has been increasing interest in the role of gut microbiota in diseases, with growing evidence demonstrating how this complex ecosystem of trillions of microorganisms actively participates in both maintaining homeostasis and driving pathogenesis across various conditions (34). Gut microbiota-derived metabolites have been demonstrated to suppress malignant tumor growth through direct and indirect interactions with cancer cells (35–38). These metabolites are consistently found at reduced levels in cancer patients. Emerging evidence suggests that such microbial metabolites can systemically influence cancer progression by remodeling the tumor microenvironment (TME) (39). The composition and activity of the TME, particularly the density and functional state of tumor-infiltrating lymphocytes (TILs), are now established as pivotal prognostic and predictive factors in cancer, forming the basis for advanced prognostic modeling (40). A growing body of research has established the significant impact of gut microbiota on PCa development, primarily mediated through microbial-derived metabolites and structural components (41). Our investigation identified significantly reduced circulating IPA levels in PCa patients, particularly those with high-grade disease. We hypothesize that this deficiency may contribute to PCa progression. Supporting this hypothesis, both *in vivo* and *in vitro* experiments demonstrated that exogenous IPA supplementation effectively inhibits PCa development.

Tumor progression is typically influenced by both antitumor immunity and intrinsic tumor cell activity (35). Prior research has indicated that IPA enhances immunotherapy efficacy by modulating T cell stemness in pan-cancer (30). Our present study, utilizing immunodeficient murine models, demonstrates that its tumor-suppressive effects are not exclusively dependent on the immune system. We observed a pronounced inhibitory effect of IPA on tumor cell activity, a finding consistently validated through both *in vitro* and *in vivo* experimental approaches. This autonomous tumor-suppressive capacity of IPA represents a complementary dimension to its previously documented immunomodulatory functions.

p53 performs multiple tumor-suppressive functions, including mediating cell cycle arrest, apoptosis, and senescence (33), and its biological activities become significantly amplified when the protein undergoes phosphorylation (42, 43). p21, as a p53-induced tumor suppressor gene (44, 45), blocks the activity of multiple cyclin-CDK complexes, resulting in hypophosphorylation of the RB protein and ultimately leading to G0/G1 cell cycle arrest (46). Our study revealed that IPA treatment upregulates protein levels of p53, phosphorylated p53, and p21, while downregulating phosphorylated RB in 22RV1 cells, indicating activation of the p53-p21-RB signaling pathway, which may potentially lead to G0/G1 cell cycle arrest. This phenomenon was confirmed by *in vitro* flow cytometric analysis. Furthermore, animal experiments revealed that IPA significantly reduced the population of Ki67-positive cells in xenograft tumors. Since Ki67 protein is rarely detected in G0 phase, but highly expressed in the nuclear region of proliferating cells (G2 and early M phases) (47), these findings indirectly substantiate the occurrence of cell cycle arrest. Additionally, elevated p53 expression promoted cellular apoptosis (33), as verified by both *in vitro* flow cytometry and immunohistochemical analysis of xenograft tumors.

Beyond the mechanistic insights into its association with the p53 pathway, our study further highlights the unique translational value of IPA. As a natural gut microbiota-derived metabolite with documented multi-system protective effects (18), IPA exhibited potent antitumor activity in our models. Notably, this efficacy was achieved in the absence of observable treatment-related systemic toxicity: all mice survived the 17-day subcutaneous administration period without significant body weight loss or overt signs of distress, indicating favorable tolerability at the effective doses. Furthermore, its low molecular weight and hydrophobic properties suggest favorable membrane permeability and potential oral bioavailability. Its physiological presence in the systemic circulation confirms its intrinsic ability to distribute throughout the body. Together, these properties position IPA as a highly promising lead compound or nutraceutical candidate. Its development may circumvent the severe side effects associated with conventional cytotoxic therapies, offering a novel and well-tolerated therapeutic strategy for prostate cancer.

While this study identifies IPA deficiency in PCa patients and demonstrates its antitumor effects, several limitations should be considered. First, the clinical relevance is constrained by our single-center cohort with a limited sample size, which may not fully capture population heterogeneity and increases the risk of false discoveries. Second, the use of non-targeted metabolomics (UPLC–MS/MS) provided relative quantitation of IPA levels, which, while suitable for identifying differences between groups, does not establish its absolute physiological concentration. Third, the immunodeficient mouse model, while validating direct antitumor activity, fails to recapitulate the complexity of the human tumor immune microenvironment. Furthermore, our mechanistic insights are derived primarily from p53 wild-type cellular models. Future studies employing p53-deficient models are essential to confirm the pathway necessity and to define IPA’s applicability across different molecular subtypes of PCa. The upstream mechanism of p53 activation by IPA also remains unclear. Finally, the clinical translation of IPA requires systematic pharmacokinetic and safety studies in humans.

## 5 Conclusion

This study establishes that IPA, a gut microbiota-derived metabolite, exerts direct antitumor effects in PCa. Our findings demonstrate that IPA treatment induces cell cycle arrest at the G0/G1 phase and promotes apoptosis in PCa cells, concurrent with the activation of the p53-p21-RB signaling axis, and significantly suppresses tumor growth *in vivo*. The identification of IPA deficiency in PCa patients, particularly those with advanced disease, suggests its potential role as both a diagnostic biomarker and a promising therapeutic agent. These results not only provide new insights into the molecular mechanisms underlying the gut-prostate axis but also highlight the potential of microbiota-based metabolic interventions as a novel strategy for PCa management. Importantly, as a natural gut microbiota-derived metabolite with a favorable safety profile, IPA itself presents significant translational advantages as a potential well-tolerated agent for PCa intervention.

## Data Availability

The raw sequence data reported in this paper have been deposited in the Genome Sequence Archive (Genomics, Proteomics & Bioinformatics 2025) at the National Genomics Data Center (Nucleic Acids Res 2025), China National Center for Bioinformation / Beijing Institute of Genomics, Chinese Academy of Sciences, and are publicly accessible at https://ngdc.cncb.ac.cn/gsa under accession number CRA040073.

## Supplementary material

Supplementary Figure 1 Orthogonal Projections to Latent Structures Discriminant Analysis (OPLS-DA) **(A, B)** OPLS-DA score plot and permutation test plot, showing clear separation between the BPH and PCa groups.
