# Supplementary figures and images for: Indole-3-propionic acid suppresses prostate cancer by inducing cell cycle arrest and apoptosis associated with p53 activation
Source: Front Oncol. 2026 Mar 25;16:1759301. doi: 10.3389/fonc.2026.1759301 (PMC13057442; doi:10.3389/fonc.2026.1759301)

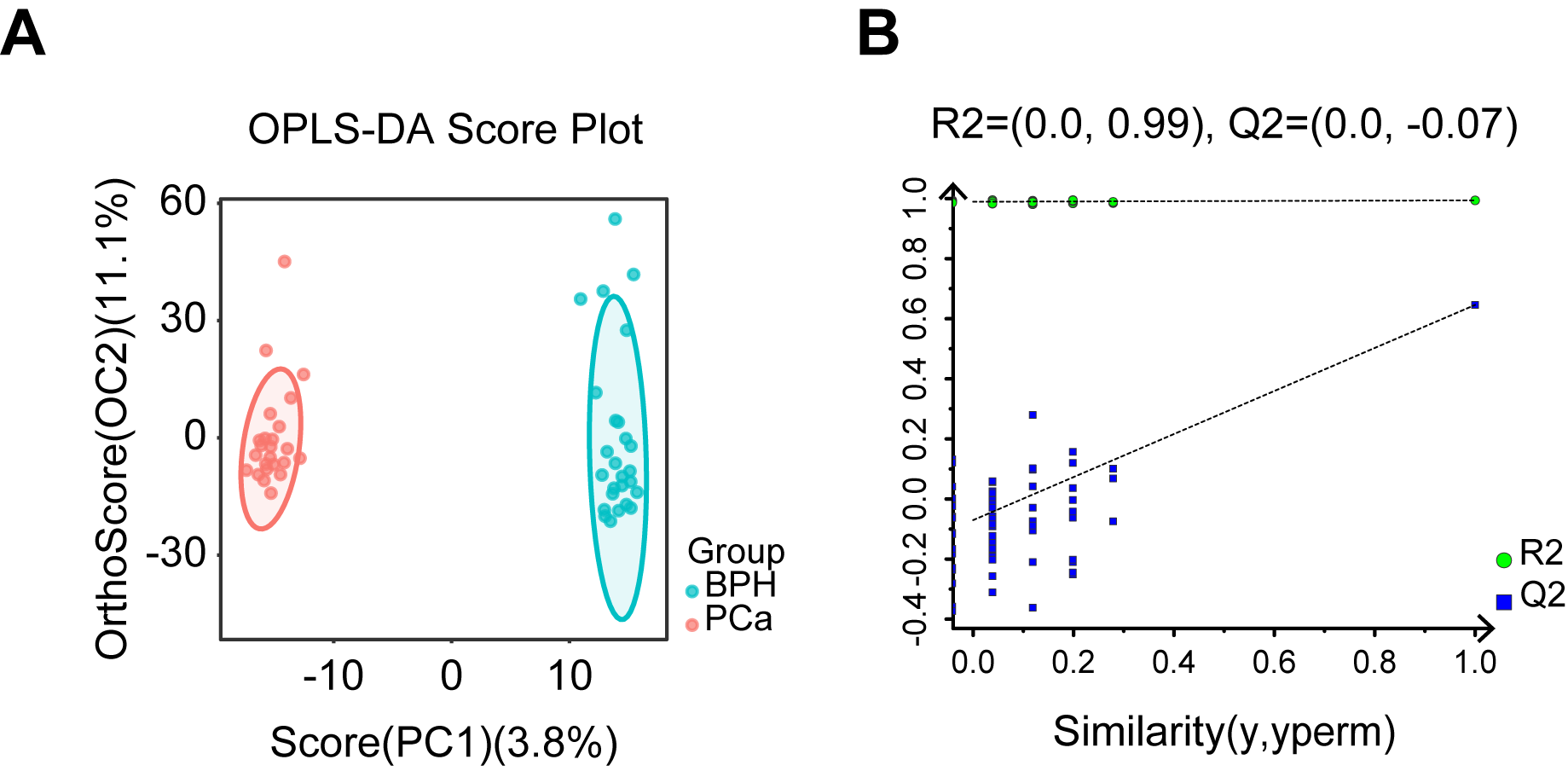

Supplement: Supplementary Figure 1 — Orthogonal Projections to Latent Structures Discriminant Analysis (OPLS-DA) (A, B) OPLS-DA score plot and permutation test plot, showing clear separation between the BPH and PCa groups. [file Image1.tif]
